# Use of machine learning to assess the prognostic utility of radiomic features for in-hospital COVID-19 mortality

**DOI:** 10.1038/s41598-023-34559-0

**Published:** 2023-05-05

**Authors:** Yuming Sun, Stephen Salerno, Xinwei He, Ziyang Pan, Eileen Yang, Chinakorn Sujimongkol, Jiyeon Song, Xinan Wang, Peisong Han, Jian Kang, Michael W. Sjoding, Shruti Jolly, David C. Christiani, Yi Li

**Affiliations:** 1 Department of Biostatistics, University of Michigan, 1415 Washington Heights, Ann Arbor, MI 48109 USA; 2 Department of Environmental Health and Epidemiology, Harvard T. H. Chan School of Public Health, 677 Huntington Avenue, Boston, MA 02115 USA; 3 Division of Pulmonary and Critical Care, Department of Internal Medicine, University of Michigan Medical School, 1500 East Medical Center Drive, Ann Arbor, MI 48109 USA; 4 Department of Radiation Oncology, University of Michigan Rogel Cancer Center, 1500 East Medical Center Drive, Ann Arbor, MI 48109 USA; 5 Division of Pulmonary and Critical Care, Department of Internal Medicine, Massachusetts General Hospital, 55 Fruit Street, Boston, MA 02114 USA

**Keywords:** Statistics, Viral infection, Outcomes research

## Abstract

As portable chest X-rays are an efficient means of triaging emergent cases, their use has raised the question as to whether imaging carries additional prognostic utility for survival among patients with COVID-19. This study assessed the importance of known risk factors on in-hospital mortality and investigated the predictive utility of radiomic texture features using various machine learning approaches. We detected incremental improvements in survival prognostication utilizing texture features derived from emergent chest X-rays, particularly among older patients or those with a higher comorbidity burden. Important features included age, oxygen saturation, blood pressure, and certain comorbid conditions, as well as image features related to the intensity and variability of pixel distribution. Thus, widely available chest X-rays, in conjunction with clinical information, may be predictive of survival outcomes of patients with COVID-19, especially older, sicker patients, and can aid in disease management by providing additional information.

## Introduction

COVID-19 has resulted in more than eighty-five million cases and over one million deaths in the United States^1^. With ongoing concerns of future resurgences^2,3^, and in an effort to improve the treatment and management of infected patients, principled methods for risk stratification and survival prognostication are critically important^4,5^. Early reports outlined diagnostic guidance for assessing chest X-ray abnormalities in emergency department settings, including patchy or diffuse reticulonodular ‘ground-glass’ opacities (GGOs) and consolidation with basal, peripheral, and bilateral predominance^6,7^. Recent studies advocated for the use of chest X-rays in grading patients with COVID-19^8,9^ via scoring systems such as the COVID-specific Brixia score, which rates lung involvement on a scale from 0 to 18, or percentage of lung involvement^10–12^. While automated approaches for disease classification have attained a high (> 90%) diagnostic accuracy^13^, there is a dearth of research using radiomic features to predict clinical outcomes for patients admitted with COVID-19 due to their high-dimensional and heterogeneous nature, as well as data unavailability^14^. The added utility of such features for predicting in-hospital mortality, beyond clinical risk factors, is largely unknown^14^.

The University of Michigan Health System (or Michigan Medicine), as one of the primary regional centers managing the care of patients with COVID-19 during the pandemic, has collected a wealth of X-ray image data, in addition to demographic and clinical data, via the Electronic Health Record (EHR)^15,16^. Portable chest X-ray, with its availability and ease of use, has been routinely used for monitoring patients in need of urgent care at Michigan Medicine, even prior to the pandemic^17^. However, analysis of chest X-ray images is complicated by the data structure, particularly in the COVID-19 setting^18^. Leveraging machine learning techniques, we proposed a workflow for the extraction and selection of features from COVID-related X-ray images. By using survival information directly, our framework decomposes raw images into texture features and identifies those features that are most related to COVID-19 mortality. We used several machine learning techniques to assess the predictability of demographic and clinical factors and the radiomic texture features on in-hospital mortality, a primary endpoint for patients hospitalized with COVID-19^19^. Subgroup analyses revealed that chest X-ray images offered more prognostic utility for vulnerable (e.g., older or sicker) patients.

## Results

### Patient outcomes and characteristics

Of the 3313 hospitalized patients with X-rays, we analyzed a total of 3310 patients with anterior–posterior or posterior-anterior images, which provided clear views of the lungs; excluded were only three patients whose X-ray provided unclear views and could not be analyzed. During follow-up, we observed 590 (17.8%) in-hospital deaths and 20 (0.6%) discharges to hospice. Median age was 61 (interquartile range: 46–73) years, and the majority of patients were male (56%), with an over-representation of Black patients (21%) as compared to the surrounding population. Median respiratory rate was 18.8 (17.5–21.7) breaths per minute and median oxygen saturation was 95.5% (94.0–97.2%). There was a high proportion of patients with cardiac arrhythmias (70%), hypertension (70%), and fluid and electrolyte disorders (70%) at admission (Supplement E). Seven radiomic features and seven clinical features were included in the final model.

### Prediction performance

We first compared the predictive performance of the following five algorithms using the clinical predictors only. The algorithms were the Cox proportional hazards model^20,21^, survival support vector machines^22^, random survival forests^23^, survival gradient boosting^24^, and ensemble averaging of the first four algorithms^25^. The average C-index across one hundred experiments ranged from 78.1 to 80.3%, with ensemble averaging performing the best. We then compared the algorithms using both the clinical and radiomic features and noted that ensemble averaging still outperformed the other methods, again achieving the highest average C-index of 81.0%. Moreover, incremental improvements were observed across all five algorithms, ranging from a 0.5% increase in C-index (random survival forests) to a 2.0% increase (survival gradient boosting; Table 1). This motivated us to conduct subgroup analyses to examine which subgroups would benefit more with the added image features; see the later section of “Subgroup analysis and risk stratification”.

**Table 1:** Comparisons of the prediction performance in concordance index between using clinical only and clinical plus imaging data, obtained by five machine learning algorithms.

Method	Clinical features only	Clinical and imaging feature
Cox proportional hazards model	78.3 (77.9, 78.7)	79.1 (78.8, 79.5)
Survival support vector machines	78.1 (77.7, 78.4)	79.6 (79.2, 79.9)
Random survival forests	79.5 (79.1, 79.9)	80.0 (79.7, 80.4)
Survival gradient boosting	78.4 (78.0, 78.8)	80.4 (80.0, 80.7)
Ensemble averaging	80.3 (79.9, 80.6)	81.0 (80.7, 81.3)

### Feature importance

**Figure 1:**
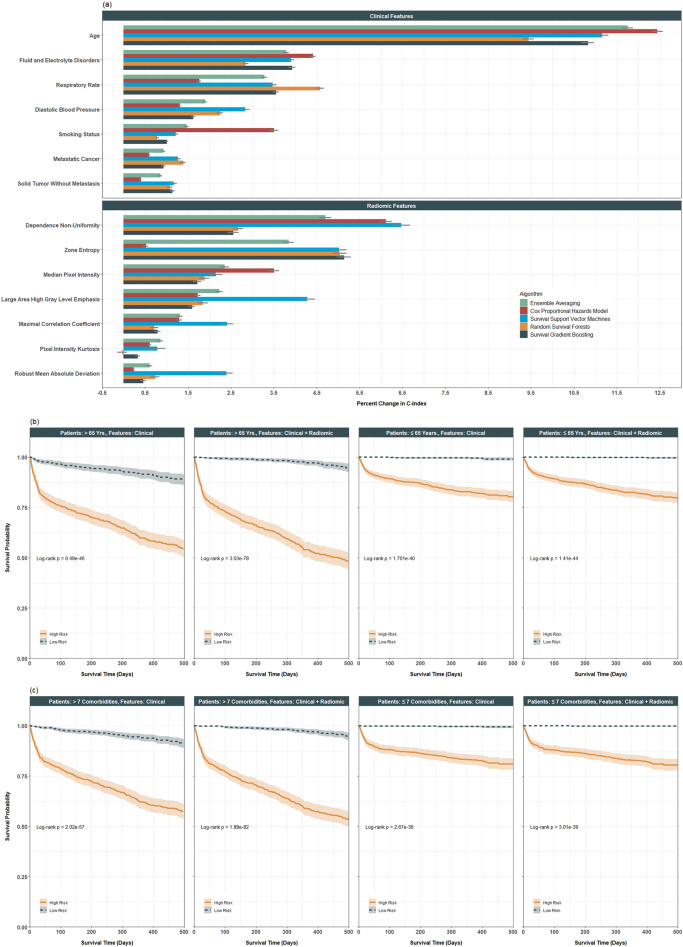
Results from predictive analysis of in-hospital mortality. (**a**) Average feature importance of clinical and imaging features based on one hundred testing datasets with standard errors, sorted by highest feature importance in ensemble averaging. (**b**) Kaplan–Meier curves for in-hospital mortality, stratified by patient age and risk group (defined by the median risk score; high risk = solid, low risk = dashed); risk scores defined either by clinical or clinical plus imaging features within each age group. (**c**) Kaplan–Meier curves for in-hospital mortality, stratified by comorbidity burden and risk group (defined by the median risk score; high risk = solid, low risk = dashed); risk scores defined either by clinical or clinical plus imaging features within each comorbidity burden group.

Figure 1a gives the feature importance for the top clinical and imaging features under the five predictive approaches. The most important clinical features were age, indications of fluid and electrolyte disorders, respiratory rate, diastolic blood pressure, metastatic cancer, and solid tumor cancer without metastasis. Important imaging texture features included dependence non-uniformity, zone entropy, median pixel intensity, large area high gray level emphasis, maximal correlation coefficient, pixel intensity kurtosis, and robust mean absolute deviation. Patients with higher dependence non-uniformity, zone entropy, and maximal correlation coefficients had more heterogeneity or complexity in the texture patterns for their images. Those with higher median pixel intensity and large area high gray level emphasis had greater concentrations of high gray level values in their images, and those with higher pixel intensity kurtosis and robust mean absolute deviations had more outlying values in their pixel intensities^26^.

### Adjusted associations between clinical/imaging features and survival

We fit a Cox regression model with the important features, presenting the hazard ratios (HR) and 95% confidence intervals (CI) in Table 2. Older age (HR: 2.33; 95% CI 2.07–2.63), higher respiratory rate (1.41; 1.28–1.55), and indications of fluid and electrolyte disorders (2.57; 1.98–3.34), metastatic cancer (1.41; 1.10–1.80), and solid tumor cancer without metastasis (1.32; 1.03–1.68) were significantly associated with higher in-hospital mortality. Conversely, higher diastolic blood pressure (0.81; 0.75–0.88), never smoking (0.46; 0.32–0.67) and former smoking (0.62; 0.43–0.90) were associated with lower mortality. Among the radiomic texture features, greater dependence non-uniformity (1.21; 1.08–1.36), large area high gray level emphasis (1.14; 1.04–1.25), and median pixel intensity (1.14; 1.05–1.25) were significantly associated with higher hazards for mortality, while lower maximal correlation coefficients (0.91; 0.83–0.99) were marginally associated with higher mortality hazards.

**Table 2:** Adjusted associations from a Cox proportional hazards model fit on the selected clinical and imaging features taken on the n = 3310 patients hospitalized with COVID-19 in our study population.

	HR	95% CI	p-value
**Clinical features**			
Age	2.33	(2.07, 2.63)	< 0.005
Respiratory rate	1.41	(1.28, 1.55)	< 0.005
Fluid and electrolyte disorders	2.57	(1.98, 3.34)	< 0.005
Diastolic blood pressure	0.81	(0.75, 0.88)	< 0.005
Metastatic cancer	1.41	(1.10, 1.80)	0.01
Solid tumor without metastasis	1.32	(1.03, 1.68)	0.03
Smoking status			
Current	–	–	–
Former	0.62	(0.43, 0.90)	0.01
Never	0.46	(0.32, 0.67)	< 0.005
Unknown	0.95	(0.65, 1.38)	0.79
**Imaging features**			
Dependence non-uniformity	1.21	(1.08, 1.36)	< 0.005
Large area high gray level emphasis	1.14	(1.04, 1.25)	0.01
Median	1.14	(1.05, 1.25)	< 0.005
Maximal correlation coefficient	0.91	(0.83, 0.99)	0.03
Robust mean absolute deviation	1.07	(0.98, 1.18)	0.14
Zone entropy	1.07	(0.95, 1.22)	0.26
Kurtosis	0.97	(0.87, 1.10)	0.66

*HR* hazard ratio, *CI* confidence interval.

### Subgroup analysis and risk stratification

We used ensemble averaging, which was the most predictive, to construct risk scores with and without the addition of the radiomic features. We compared how these scores could distinguish patients within certain subgroups, defined by age or comorbidity burden (Fig. 1b,c). Two findings are worth noting. First, the scores, based on clinical indicators only or both clinical and image features, could well distinguish patients across all the subgroups, highlighting the usefulness of clinical and image features in profiling the risk of patient mortality.

Second, patients were then classified as ‘high’ versus ‘low’ risk based on median risk scores defined by using both clinical and clinical + radiomic features. Within certain subgroups (e.g., patients older than 65 years or those with seven or more comorbid conditions), the separation between the survival curves of the high- and low-risk patients defined with the addition of the imaging features was more obvious than that between those of the high- and low-risk patients, defined using clinical features alone. This exemplifies the added prognostic utility of radiomic features in these subgroups. In contrast, the separation was not as apparent in the other subgroups, e.g., among those younger than 65 years or with fewer than seven comorbidity conditions.

To confirm our findings, we compared the increase in C-index with the addition of the radiomic features between these different subgroups. Table 3 shows a significantly higher increase in C-index among older patients than younger patients with the addition of radiomic features. There was a 2.3–3.1% increase in C-index among older patients across the different algorithms with the addition of the radiomic features. This increment is clinically meaningful^27,28^ and significantly larger (p < 0.001) than the 0.5–1.0% increase among younger patients. Similarly, a 1.6–2.5% increase in C-index was achieved among patients with a higher comorbidity burden, as compared to a 0.2–1.4% increase among patients with a lower comorbidity burden. This increment was clinically meaningful and statistically significant (p < 0.01).

**Table 3:** Prediction performance in concordance index of different algorithms comparing (1) patients 65 years or younger versus older than 65 years, and (2) patients with seven or fewer versus more than seven comorbidities.

Method	Age ≤ 65 (n = 1976)			Age > 65 (n = 1334)			p-value
	Clinical	Clinical and imaging	Improvement	Clinical	Clinical and imaging	Improvement	
Cox proportional hazards model	79.1 (78.5, 79.7)	79.7 (79.1, 80.3)	0.6 (0.4, 0.8)	69.2 (68.5, 69.9)	71.5 (71.0, 72.0)	2.3 (1.9, 2.7)	< 0.001
Survival support vector machines	79.6 (79.1, 80.1)	80.3 (79.8, 80.8)	0.7 (0.5, 0.9)	69.3 (68.7, 69.9)	72.3 (71.8, 72.8)	3.0 (2.6, 3.4)	< 0.001
Random survival forests	79.0 (78.4, 79.6)	80.0 (79.5, 80.5)	1.0 (0.6, 1.4)	69.7 (69.1, 70.3)	72.8 (72.2, 73.4)	3.1 (2.6, 3.6)	< 0.001
Survival gradient boosting	79.1 (78.6, 79.6)	80.1 (79.5, 80.7)	1.0 (0.5, 1.5)	70.1 (69.5, 70.7)	73.2 (72.7, 73.7)	3.1 (2.6, 3.6)	< 0.001
Ensemble averaging	80.6 (80.1, 81.1)	81.1 (80.6, 81.6)	0.5 (0.2, 0.8)	71.1 (70.5, 71.7)	73.6 (73.1, 74.1)	2.5 (2.1, 2.9)	< 0.001

Method	≤ 7 Comorbidities (n = 1679)			> 7 Comorbidities (n = 1631)			p-value
	Clinical	Clinical and imaging	Improvement	Clinical	Clinical and imaging	Improvement	
Cox proportional hazards model	82.9 (82.3, 83.5)	83.1 (82.5, 83.7)	0.2 (0.0, 0.4)	71.3 (70.8, 71.8)	72.9 (72.4, 73.4)	1.6 (1.4, 1.8)	< 0.001
Survival support vector machines	83.3 (82.8, 83.8)	83.6 (83.0, 84.2)	0.3 (0.0, 0.6)	71.1 (70.6, 71.6)	73.2 (72.7, 73.7)	2.1 (1.7, 2.5)	< 0.001
Random survival forests	82.2 (81.6, 82.8)	83.6 (83.1, 84.1)	1.4 (1.0, 1.8)	70.4 (69.9, 70.9)	72.5 (72.0, 73.0)	2.1 (1.7, 2.5)	0.01
Survival gradient boosting	82.2 (81.6, 82.8)	82.8 (82.1, 83.5)	0.6 (0.1, 1.1)	70.6 (70.1, 71.1)	73.1 (72.6, 73.6)	2.5 (2.1, 2.9)	< 0.001
Ensemble averaging	84.2 (83.7, 84.7)	85.0 (84.5, 85.5)	0.8 (0.5, 1.1)	72.0 (71.5, 72.5)	74.2 (73.7, 74.7)	2.2 (1.9, 2.5)	< 0.001

## Discussion

Many recent studies have discussed the potential of integrative models for discovery and prognostication in a wide range of clinical settings, including breast^29^ and lung^30^ cancers, coronary artery disease^31^, and pulmonary embolisms^32^. Each of these studies have demonstrated that multimodal prediction methods, which combine radiomic and clinical features, allow for improved predictive performance in a range of clinical settings. Further, recent works have suggested radiomic phenotypes from medical imaging are linked with molecular phenotypes, such as genomics and histopathology, and therefore may provide important and clinically relevant information^33,34^. In our setting, radiologic imaging plays an important role in grading and managing patients with COVID-19, as portable chest X-rays are an efficient and convenient means of triaging emergent cases. This work addresses the question as to whether imaging carries any additional prognostic utility in the management of patients with COVID-19. We observed a slight increase in prediction performance with the added X-ray features, which motivated us to further study which patient subgroups would benefit more from the additional image features.

Across all patients, we saw modest improvements in the predictive accuracy of the methods under comparison, but we found that older patients and those with higher comorbidity burden at admission saw significantly larger gains in C-index with the added radiomic features. Though the magnitudes of these improvements are relatively small, we recognize the potential clinical impact. Even a slight increase in prediction accuracy could benefit patient outcomes by helping clinicians identify high-risk patients and initiate timely interventions. Not every patient’s physiological derangements correlate closely with their images^35,36^; for instance, younger patients with bilateral peripheral pulmonary infiltrates can be oxygenating well and appear fine clinically, whereas older or sicker patients may be less able to compensate for the same degree of imaging abnormalities. Hence, while the metrics identified in our study may not be useful in every case, they may help pinpoint which patient subpopulations can more reliably benefit from imaging as a predictor of mortality.

There is a growing body of literature to support the use of imaging data for in-hospital mortality prognostication. Kim et al. found that X-ray grade was significantly associated with both length of stay in hospital and higher odds of intubation^7^. Garrafa et al. predicted in-hospital mortality using the COVID-specific Brixia score^37^, and the predictiveness for their testing data ranged from 0.52 (logistic regression) to 0.78 (random forests and gradient boosting), which was close to our results. Schalekamp et al. graded chest X-rays on a severity scale from zero to eight points^38^ and developed an image-based risk score to predict critical illness in patients with COVID-19. Soda et al. modeled patient survival with clinical and imaging features in an Italian cohort^39^ and obtained an accuracy of 0.68–0.76 across different methods with only clinical information and increasing to a range of 0.75–0.77 with both clinical and imaging features, an increment similar to our report. They found that age, oxygen saturation, respiratory rates, and active cancer were of the most importance, which was consistent with our findings.

Lung involvement and COVID-19 severity, assessed by visual examination of the raw X-ray images, were reported to be predictive of mortality^10–12,40^. However, visual approaches may be prone to subjectivity and inaccuracy. Recent works have shown that texture features associated with image heterogeneity are predictive of clinical outcomes when visual assessment of imaging data may not be correlated with these same endpoints^41^. In contrast, our method provides an objective means of extracting image features for aiding in survival prognostication. Our work also addresses the challenge of analyzing variable-size images, which cannot be processed by deep learning algorithms like AlexNet^42^ or ResNet^43^. Rather than directly feeding images into the models, we derived relevant texture features with maximal image differentiation for predicting COVID-19 survival based on a standard workflow^13,14,44–47^. These texture features may also be more interpretable than those derived from deep learning models^48^.

Further, our method enabled us to leverage patient survival information when selecting the image features, leading to some interesting discoveries. We found that median pixel intensity and large dependence high gray level emphasis, features corresponding to greater concentrations of high gray level values in the images, were important predictors of patient survival. Greater heterogeneity in the texture features, characterized by zone entropy and dependence non-uniformity was also predictive. These findings align with the current literature. For example, similar to our results, Varghese et al. showed the importance of certain first and second order texture features, namely, histogram and intensity, followed by the gray level size zone matrix and grey level co-occurrence matrix, for predicting intensive care unit utilization, intubation, and death^49^. Iori et al. identified important texture features, including entropy and dependence non-uniformity, for mortality prediction^50^.

We detected that predictions on certain subgroups of patients benefited more from the addition of these radiomic features. In particular, greater improvement in survival prediction was observed for older (> 65 years) patients and those with higher (> median 7/29 comorbidities) comorbidity burden. Our results agree with previous findings that the severity of disease in the images is associated with comorbidity burden and age^51–53^, hinting that radiomic features coming from older or sicker patients are likely to contain more information relevant to survival. In contrast, younger or healthier patients are at a lower risk of death, so the additional radiomic features do not add much to their prognostication^54^.

We note some limitations and areas of future work for the current study. First, only hospitalizations at Michigan Medicine were included in the analysis, potentially limiting the generalizability of the results. However, our workflow provides a general and useful framework for analyzing EHR data with chest X-ray images, and our results may generate hypotheses for larger-scale investigations, and potentially in other disease areas as well. As some improvement was observed among older patients and patients with a higher comorbidity burden, external validation is necessary to confirm these results and their clinical importance. Further investigations are also needed to assess the optimality of our feature extraction and screening techniques and the predictive accuracy of our approach as compared to standard clinical practice. We selected clinical predictors which were known to be predictive of worsened COVID-19 outcomes based on clinical practice and the current body of literature. However, risk factors which are viewed as important may differ across institutions with varying and complex patient populations. Further, to assess whether our proposed computer-based multi-modal approach can augment the practice of medicine, a next step would be to design a study in which predictions generated from our model are compared to those generated from clinicians based on a common a set of imaging and clinical features. This focus group approach would be an important next step in the continuation and validation of the current work. Lastly, comparisons to other, automated approaches such as deep learning may yield additional benchmarks for the accuracy of the proposed method.

Second, as with most EHR studies, there might be an inherent selection bias among those presenting to Michigan Medicine and subsequently admitted for COVID-19 related complications. Causal inference approaches may be explored to address observable and unobservable confounders. Third, comorbidities taken at admission were not differentiated from chronic conditions preceding infection. More in-depth work is needed to account for chronologies of these conditions. Moreover, comorbid conditions were established via IDC-10 codes based on Quan et al.^55^. At our institution, diagnoses with associated ICD-10 codes are typically first input into the EHR on admission and later confirmed retrospectively. However, there may be administrative delays in the coding of these conditions after a patient's initial encounter, both in our study and broadly at other institutions. This may require adequate alternatives for comorbidity identification, particularly for those conditions included in our final model. For example, the patients in our study population who were broadly indicated for fluid and electrolyte disorders had imbalances that span the range of sodium and potassium, acidosis, alkalosis, and volume depletion which coincide with both each other and worsened COVID-19 outcomes. An alternative strategy for identifying such conditions would be in flagging patients based on abnormal blood and urine laboratory values, e.g., increased urine sodium and/or osmolality, which may be more readily available. As the current method stands, however, this is a potential limitation for the immediate useability of the method in other practical clinical settings.

Lastly, mortality is often a key endpoint for identifying patients who are at high risk of adverse events or who may need closer monitoring and more aggressive interventions. However, as COVID-19 has a wide range of presentation, bearing many clinical abnormalities, mortality should not be used in isolation, and additional patient centered outcomes, such as the patient-specific quality of life and care, should be considered when making clinical decisions^56,57^.

## Conclusions

In summary, portable chest X-ray is a valuable tool for monitoring and guiding the care of patients with COVID-19. This study found that patterns of COVID-19 lung disease identified on chest X-ray are predictive of, and significantly associated with, the survival outcomes of patients hospitalized with COVID-19. Multimodal prediction models may provide modest improvements in prognostic value over clinical risk factor alone, and further research into understanding the clinical and biological underpinnings of these improvement are necessary to provide additional information when guiding the management of patients with COVID-19.

## Methods

### Experimental design

This was a prognostic analysis of patients who (1) were admitted to Michigan Medicine between March 10, 2020 (the date of the first case in this state) and March 31, 2022 (the cutoff date of the released EHR data), (2) tested positive for COVID-19 or transferred in carrying a positive diagnosis, and (3) had at least one COVID-related chest X-ray image taken. We focused on patients with X-rays because patients without imaging were in general much younger and healthier, and images are valuable in triaging patients and managing resources^58^. Our outcome was the time from admission until in-hospital death, censored by discharge or the end of the study. Discharge was regarded as a censoring event, except for discharge to hospice, because the median survival for these patients was less than 30 days post-discharge. As it was a strong precursor to death, we considered both in-hospital death and discharge to hospice as failure events (see Supplement A).

From the EHR database, we extracted and created a set of demographic, socioeconomic, and clinical risk factors (see Supplement B) identified as being related to COVID-19 in the literature^59–72^. Patient demographics included age, sex, race (Black or non-Black), ethnicity (Hispanic or non-Hispanic), smoking status, alcohol use, and drug use. As patient-level socioeconomic factors were unavailable, we created four composite socioeconomic measures at the US census tract-level based on patient residences. These composites, measuring affluence, disadvantage, ethnic immigrant concentration, and education, were defined to be the proportion of adults meeting the corresponding criterion within a census tract^73–75^, and were further categorized by quartiles. For each of twenty-nine prevalent comorbidity conditions commonly used in literature^55,76–78^, we defined a binary indicator to flag whether the patient had any associated ICD-10 code at admission. Lastly, we obtained physiologic measurements within 24 h of admission, including body mass index (kg/m^2^), oxygen saturation, body temperature, respiratory rate, diastolic and systolic blood pressure, and heart rate.

**Figure 2:**
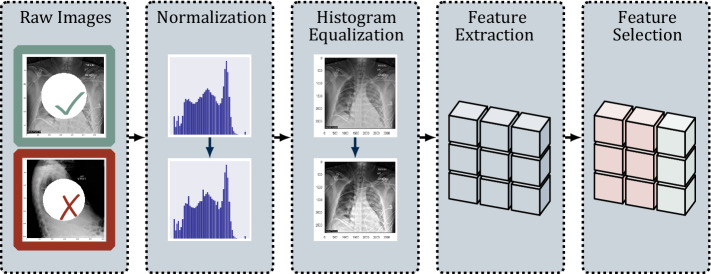
Image pre-processing procedure. Flowchart of pre-processing steps from (1) raw image selection, (2) pixel normalization, (3) histogram equalization, (4) feature extraction, and (5) feature selection.

With multiple X-rays potentially taken for one patient, we chose the one closest to the time of admission and examined its role in predicting patient survival. We first pre-processed each image according to the pipeline depicted in Fig. 2. First, prior to feature extraction and selection, we retained only those images taken from the anterior–posterior or posterior-anterior positions so that the orientation of the images would be comparable. We then normalized these images so that the pixel intensities of each image conformed to a standard range of 0 (black) to 255 (white) units. We further used histogram equalization to enhance the contrast of the images^79^.

Broadly, there are two potential approaches for feature extraction, namely (1) artificial intelligence methods, which learn feature representations automatically from the data, and (2) engineered texture features. While deep learning has been shown to have high prognostic accuracy, learned features are difficult to interpret, not standardized, and often not reproducible, which may impact their reliability^80^. Thus, we extracted a standard panel of engineered texture features according to the PyRadiomics workflow^47,81^. Specifically, we applied six different filters (e.g., different transformations) to the pre-processed images to acquire additional information (e.g., at edges or boundaries) and derive different image types (e.g., shape)^47^. From the seven image filters (original + six transformations), we extracted seven classes of features from each image^47,82–84^, resulting in 1311 candidate image features. To obtain a short list of predictive clinical and image features, we performed feature screening by fitting Cox proportional hazards models^21^ on each feature one at a time and retaining those significant at the 0.05 level. Finally, we selected the features with the highest feature importance, and obtained a final Cox model, quantifying the adjusted associations of important clinical and radiomic features with in-hospital mortality^85^. We used the concordance index (C-index) to assess the predictiveness of models^27,28^ (see Supplement C). This study was approved by the Michigan Medicine Institutional Review Board (HUM00192931), which waived informed consent based on secondary analysis of deidentified datasets. All analysis was conducted in accordance with relevant guidelines and regulations.

### Statistical analysis

We implemented five risk prediction algorithms, namely, the Cox proportional hazards model^21^, survival support vector machines^22^, random survival forests^23,86^, survival gradient boosting^24^, and ensemble averaging of the first four algorithms^25^. The Cox model, the most widely used method in survival analysis, assumes a risk function that is linear in the predictors. Survival support vector machines^22^ can account for non-linear relationships. Both random survival forests and survival gradient boosting combine multiple predictions from individual survival trees to achieve a more powerful prediction^23,24,86,87^. Ensemble averaging combines predictions from multiple models to produce a desired output and often performs better than individual models by averaging out their errors^25^. Supplement D details these methods.

We used cross-validation to unbiasedly estimate the predictiveness of each method. We randomly split the data into 80% training and 20% testing samples, maintaining the proportion of events in the full sample within each split. We then trained the various predictive models by using the training samples and computed the C-index by using the testing samples. We repeated the same procedure one hundred times and took an average of the C-index to obtain an unbiased estimate of the C-index for each method^88,89^. We applied each method with the demographic and clinical predictors, followed by the addition of radiomic features to assess their incremental prognostic utility via the C-index. Using ensemble averaging, which was the most predictive (see the section of “Results”), we developed a risk score to predict in-hospital mortality and classified patients into low- and high-risk groups using the median score as the cutoff.

Lastly, we detail the variable selection process for building a final Cox model. We selected clinical and image features based on their importance in prediction, defined by the absolute decrease in C-index with the “removal” of the concerned feature in the data^90^. To do so, we randomly split the data into 80% training and 20% testing samples, fit the model on the training data and calculated the feature importance using the testing data (Supplement D.6). We repeated the same procedure one hundred times, selected the features that were most important (on average) among these one hundred experiments, and included them in a multivariable Cox regression to assess their statistical associations with in-hospital mortality. All data processing and analysis was carried out with Python (version 3.8.8), NumPy (version 1.20.1), and scikit-survival (version 0.17.2).

We examined different subgroups to gauge how the prediction performance of the model improved with the added radiomic features. Because age and comorbidity burden were the most relevant to survival among the clinical factors, we considered patient subgroups defined by age (> versus ≤ 65 years old) and number of comorbidities at admission (> versus ≤ median seven comorbidities), respectively. We compared the change in prediction performance with the addition of the radiomic features between different subgroups.

### Ethics approval

This study was approved by the Michigan Medicine Institutional Review Board (HUM00192931), which waived informed consent based on secondary analysis of deidentified datasets. All analysis was conducted in accordance with relevant guidelines and regulations.

## Supplementary Information

Supplementary Information.

## Data Availability

The datasets used in this study are not publicly available due to the need for institutional review board approval as a University of Michigan-affiliated researcher through the University of Michigan Health System (i.e., Michigan Medicine) Precision Health Initiative. For more information, please contact PHDataHelp@umich.edu.

## Code availability

All code used to produce the results found in this work have been made publicly available at https://github.com/YumingSun/COVID_Imaging_Prediction.
