# Supplementary material for: Use of machine learning to assess the prognostic utility of radiomic features for in-hospital COVID-19 mortality
Source: Sci Rep. 2023 May 5;13:7318. doi: 10.1038/s41598-023-34559-0 (PMC10161188; doi:10.1038/s41598-023-34559-0)
Supplement: Supplementary file 1 — Supplementary Information. [file 41598_2023_34559_MOESM1_ESM.docx]

**Supplemental Materials**

This document provides supplemental materials for the manuscript, entitled *"Use of Machine Learning to Assess the Prognostic Utility of Radiomic Features for In-hospital COVID-19 Mortality.”*

**Authors**

Yuming Sun^1†^, Stephen Salerno^1†^, Xinwei He^1^, Ziyang Pan^1^, Eileen Yang^1^, Chinakorn Sujimongkol^1^,

Jiyeon Song^1^, Xinan Wang^2^, Peisong Han^1^, Jian Kang^1^, Michael W Sjoding^3^, Shruti Jolly^4^,

David C Christiani^2,5^, and Yi Li^1*^

^1^Department of Biostatistics, University of Michigan, 1415 Washington Heights, Ann Arbor, 48109, MI, United States.

^2^Department of Environmental Health and Epidemiology, Harvard T. H. Chan School of Public Health, 677 Huntington Avenue, Boston, 02115, MA, United States.

^3^Division of Pulmonary and Critical Care, Department of Internal Medicine, University of Michigan Medical School, 1500 East Medical Center Drive, Ann Arbor, 48109, MI, United States.

^4^Department of Radiation Oncology, University of Michigan Rogel Cancer Center, 1500 East Medical Center Drive, Ann Arbor, 48109, MI, United States.

^5^Division of Pulmonary and Critical Care, Department of Internal Medicine, Massachusetts General Hospital, 55 Fruit Street, Boston, 02114, MA, United States.

^*^Corresponding author(s). E-mail(s): [yili@umich.edu](mailto:yili@umich.edu);

Contributing authors: [yumsun@umich.edu](mailto:yumsun@umich.edu); [salernos@umich.edu](mailto:salernos@umich.edu); [xinweih@umich.edu](mailto:xinweih@umich.edu); [ziyangp@umich.edu](mailto:ziyangp@umich.edu); [ejyang@umich.edu](mailto:ejyang@umich.edu); [chinaks@umich.edu](mailto:chinaks@umich.edu); [jiyeons@umich.edu](mailto:jiyeons@umich.edu); [xinanwang@hsph.harvard.edu](mailto:xinanwang@hsph.harvard.edu); [peisong@umich.edu](mailto:peisong@umich.edu); [jiankang@umich.edu](mailto:jiankang@umich.edu); [msjoding@med.umich.edu](mailto:msjoding@med.umich.edu); [shrutij@med.umich.edu](mailto:shrutij@med.umich.edu); [dchris@hsph.harvard.edu](mailto:dchris@hsph.harvard.edu);

^†^These authors contributed equally to this work.

# A. Outcome Definition

Our primary outcome was in-hospital survival, defined as the time from index COVID-19 hospitalization until death, which could be censored by discharge or the end of the study window. Discharge was regarded as a censoring event, apart from discharge to hospice, which functioned as a precursor to death. As shown in Figure S1, median survival for patients discharged to hospice, whether at home or in a medical facility, was less than 30 days post-discharge. Thus, our outcome was defined as time to death or discharge to hospice, whichever came first. This composite endpoint could then be censored by discharge of any other type or administrative censoring on March 31, 2021.


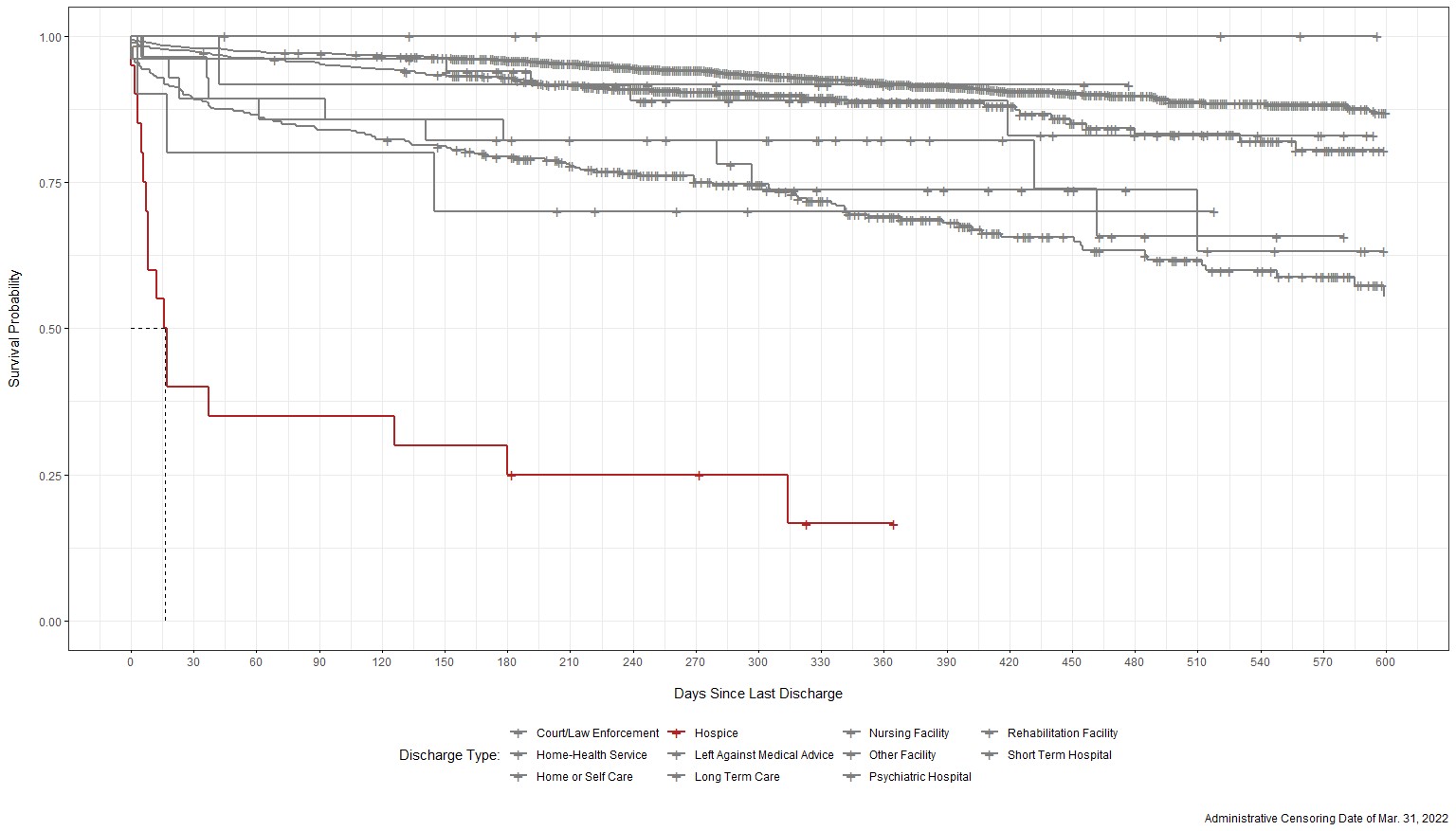


**Fig. S1**: Kaplan-Meier curves for patients discharged alive, stratified by discharge type.

# B. Demographic and Clinical Predictors

## Neighborhood Socioeconomic Status

We defined four composite measures of neighborhood socioeconomic status at the US census tract-level based on patient residences (Clarke and Melendez, Ann Arbor, MI; Gu et al, 2020; Salerno et al, 2021b). These composites, derived from the National Neighborhood Data Archive, measured a neighborhood’s (1) affluence, (2) disadvantage, (3) ethnic immigrant concentration, and (4) education, and were defined in the average proportion of adults within a census tract fall meeting each respective measure’s criteria. Each measure was aggregated and was further categorized by quartiles (Table S1).

- **Affluence:** the proportion of households with income greater than $75K, proportion of the population aged 16+ employed in professional or managerial occupations, and proportion of adults with bachelor’s degrees or higher.
- **Disadvantage:** the proportion of non-Hispanic Black, proportion of female-headed families with children, proportion of households with public assistance income or food stamps, proportion of families with income below the federal poverty level, and proportion of the population aged 16+ unemployed.
- **Ethnic Immigrant Concentration:** the proportion of Hispanic and proportion of foreign born.
- **Education:** the proportion of adults with less than a high school diploma.

## Elixhauser Comorbidity Conditions

Table S1 lists the comorbidity conditions considered as risk factors in this analysis and the corresponding ICD-10 codes used to define them. Each comorbidity was coded as a binary indicator, flagging whether a patient carried any ICD-10 code associated with the condition at baseline.

**Table S1**: Elixhauser comorbidity conditions and associated ICD-10 codes.

| Comorbidity Condition | ICD-10 Codes |
| --- | --- |
| Congestive Heart Failure | I09.9, I11.0, I13.0, I13.2, I25.5, I42.0, I42.5-I42.9, I43.x, I50.x, P29.0 |
| Cardiac Arrhythmias | I44.1-I44.3, I45.6, I45.9, I47.x-I49.x, R00.0, R00.1, R00.8, T82.1, Z45.0, Z95.0 |
| Valvular Disease | A52.0, I05.x-I08.x, I09.1, I09.8, I34.x-I39.x, Q23.0- Q23.3, Z95.2-Z95.4 |
| Pulmonary Circulation Disorders | I26.x, I27.x, I28.0, I28.8, I28.9 |
| Peripheral Vascular Disorders | I70.x, I71.x, I73.1, I73.8, I73.9, I77.1, I79.0, I79.2, K55.1, K55.8, K55.9, Z95.8, Z95.9 |
| Hypertension, Uncomplicated | I10.x |
| Hypertension, Complicated | I11.x-I13.x, I15.x |
| Paralysis | G04.1, G11.4, G80.1, G80.2, G81.x, G82.x, G83.0- G83.4, G83.9 |
| Other Neurological Disorders | G10.x-G13.x, G20.x-G22.x, G25.4, G25.5, G31.2, G31.8, G31.9, G32.x, G35.x-G37.x, G40.x, G41.x, G93.1, G93.4, R47.0, R56.x |
| Chronic Pulmonary Disease | I27.8, I27.9, J40.x-J47.x, J60.x-J67.x, J68.4, J70.1, J70.3 |
| Diabetes, Uncomplicated | E10.0, E10.1, E10.9, E11.0, E11.1, E11.9, E12.0, E12.1, E12.9, E13.0, E13.1, E13.9, E14.0, E14.1, E14.9 |
| Diabetes, Complicated | E10.2-E10.8, E11.2-E11.8, E12.2-E12.8, E13.2- E13.8, E14.2-E14.8 |
| Hypothyroidism | E00.x-E03.x, E89.0 |
| Renal Failure | I12.0, I13.1, N18.x, N19.x, N25.0, Z49.0-Z49.2, Z94.0, Z99.2 |
| Liver Disease | B18.x, I85.x, I86.4, I98.2, K70.x, K71.1, K71.3K71.5, K71.7, K72.x-K74.x, K76.0, K76.2-K76.9, Z94.4 |
| Peptic Ulcer Disease, Excluding Bleeding | K25.7, K25.9, K26.7, K26.9, K27.7, K27.9, K28.7, K28.9 |
| Lymphoma | C81.x-C85.x, C88.x, C96.x, C90.0, C90.2 |
| Metastatic Cancer | C77.x-C80.x |
| Solid Tumor without Metastasis | C00.x-C26.x, C30.x-C34.x, C37.x-C41.x, C43.x, C45.x-C58.x, C60.x-C76.x, C97.x |
| Rheumatoid Arthritis/Collagen Vascular Diseases | L94.0, L94.1, L94.3, M05.x, M06.x, M08.x, M12.0, M12.3, M30.x, M31.0-M31.3, M32.xM35.x, M45.x, M46.1, M46.8, M46.9 |
| Coagulopathy | D65-D68.x, D69.1, D69.3-D69.6 |
| Obesity | E66.x |
| Weight Loss | E40.x-E46.x, R63.4, R64 |
| Fluid and Electrolyte Disorders | E22.2, E86.x, E87.x |
| Blood Loss Anemia | D50.0 |
| Deficiency Anemia | D50.8, D50.9, D51.x-D53.x |
| Alcohol Abuse | F10, E52, G62.1, I42.6, K29.2, K70.0, K70.3, K70.9, T51.x, Z50.2, Z71.4, Z72.1 |
| Drug Abuse | F11.x-F16.x, F18.x, F19.x, Z71.5, Z72.2 |
| Psychoses | F20.x, F22.x-F25.x, F28.x, F29.x, F30.2, F31.2, F31.5 |
| Depression | F20.4, F31.3-F31.5, F32.x, F33.x, F34.1, F41.2, F43.2 |

# C. Chest X-Ray Images

## Image Pre-Processing

To extract important radiomics features from the raw X-ray images, we followed the workflow as outlined in Figure 2 in the main text. According to the anatomical coordinate system, images can be taken along the anterior-posterior, inferior-superior, and left-right axes. We selected only those images taken from the anterior-posterior or posterior-anterior position, as they were the most common. We then normalized the pixel intensities of each image to a standard range of 0 (black) to 255 (white) units. This is done so that the pixel intensities for all images used are on similar scales, facilitating comparability. The range of 0 to 255 is chosen for computational reasons, so that each pixel intensity can be stored with less memory as an unsigned integer. Then, histogram equalization was used to enhance the contrast of the images (Jain, 1989).

We extracted seven classes of features from each image: first order, shape, gray level co-occurrence matrix, gray level size zone matrix, gray level run length matrix, neighboring gray tone difference matrix, and gray level dependence matrix features (Haralick et al, 1973; Galloway, 1975; Chu et al, 1990; Thibault et al, 2013; van Griethuysen et al, 2017). For example, patients with higher gray level run emphasis, higher large dependence high gray level emphasis, and higher median pixel intensity have a greater concentrations of high gray level values in their images. For those with higher zone entropy and gray level variance, there is more heterogeneity in the texture patterns. In addition, we applied six different filters to the original images to derive different image types: wavelet, Laplacian of Gaussian, square, square root, logarithm, and exponential filters (van Griethuysen et al, 2017). Different filters apply different transformations to the image pixel density, which modify or enhance certain image properties and provide additional information (e.g., at edges or boundaries in the image). For example, the square filter takes the square of the pixel intensities and then transforms them back to the original range. The logarithm filter takes the logarithm of the absolute intensity plus one and then scales then back to original range. Negative original values are made negative again after application of filter (van Griethuysen et al, 2017). Similar to the original images, we extracted seven classes of features from each of the transformed image types, resulting in 1,311 candidate features.

We then conduct feature selection to reduce the feature dimension. For those features with correlations higher than 0.75, we retained the features with the highest concordance (C) index from the univariate Cox proportional hazards models. We then conducted forward selection on the remaining features according to their prediction power, selecting those with the highest C-Index values (Uno et al, 2011).

## Example Patient Image Features

We exemplify the image features extracted in two patients sampled from the study population. As shown, the example censored patient had a longer observed survival time, by smaller values for large dependence high gray level emphasis and median pixel intensity, meaning that this patient had lower than average proportion of larger size zones with higher gray-level values. Further, there was less than average heterogeneity in the texture patterns, given by the lower values for dependence non-uniformity and zone entropy. In contrast, the patient that was observed to die had a shorter observed time at risk, but larger than average values for the exemplified texture features.

**Table S2**: Important texture features for two example patients.

| Measure | 1. Censored | 1. Death Observed |
| --- | --- | --- |
| Observed Survival Time (days) | 567 | 104 |
| Dependence Non-Uniformity | -0.74 | -0.61 |
| Large Area High Gray Level Emphasis | -0.70 | -0.43 |
| Median | -0.61 | 2.84 |
| Zone Entropy | -1.06 | 1.11 |

1. Censored (b) Death Observed


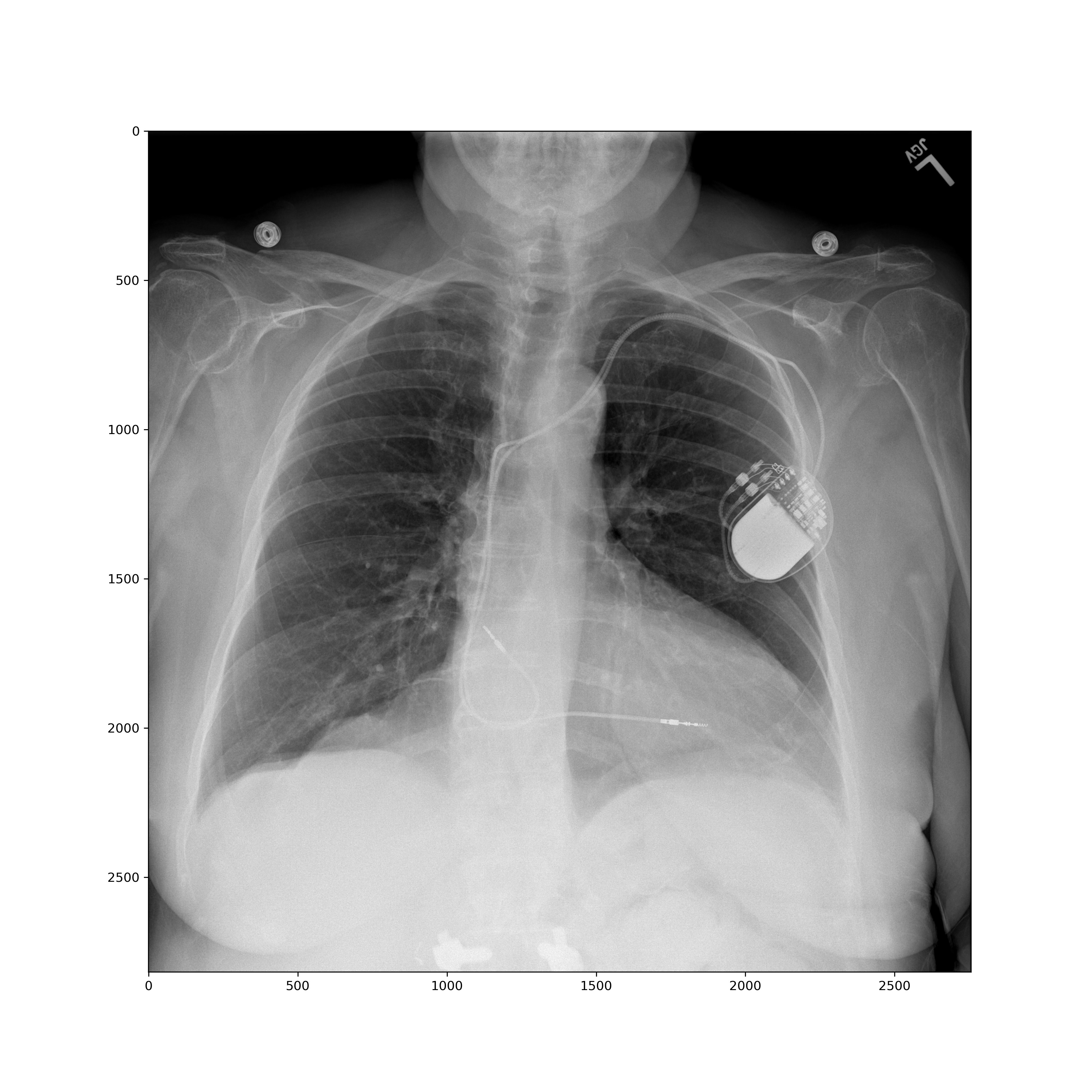

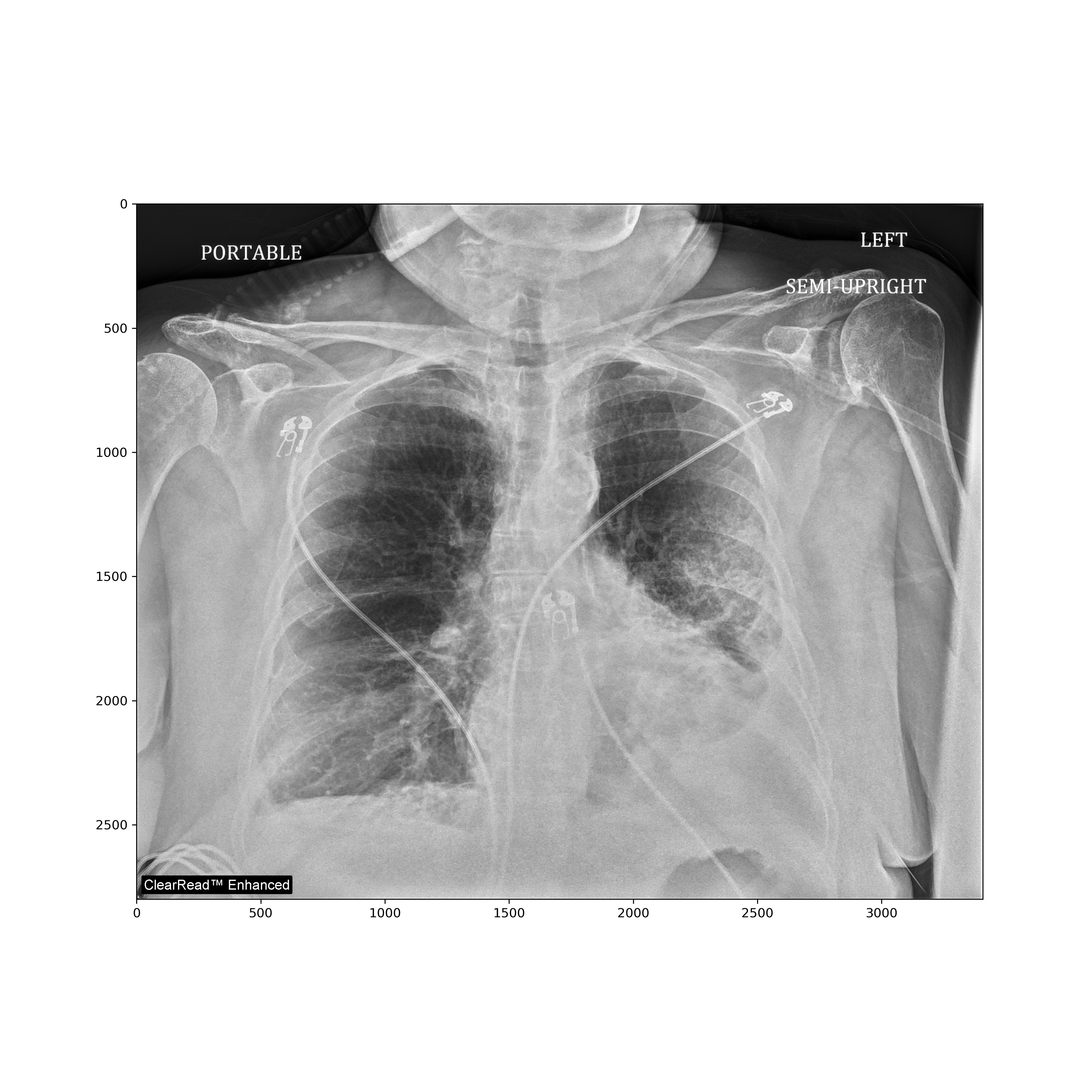


**Fig. S2**: Chest X-ray images for two example patients: (a) censored, and (b) death observed.

# D. Statistical Methods and Models

## Cox Proportional Hazards Regression

Cox proportional hazards models stipulate that the conditional hazard of in-hospital mortality given the predictors, $\boldsymbol{X}$, is

$$\lambda\left( t | \boldsymbol{X} \right)=\lambda_{0}\left( t \right)\exp\left( \boldsymbol{X}^{T}\beta\right),$$

where the baseline hazard, $\lambda_{0}(t)$, is unspecified, and $\beta=\left( \beta_{1},...,\beta_{p} \right)^{T}$ is the coefficient vector of $\boldsymbol{X}$ to be estimated. The coefficients represent the change in the log hazard ratio relative to a one-unit change in the predictor, holding all other predictors fixed (Cox, 1972).

## Support Vector Machines

In our setting, the survival support vector machine is formulated based on the rank concordance between the predicted risk and the observed survival time among comparable individuals, which is equivalent to minimizing Harrell’s C-index (Van Belle et al, 2007; Harrell et al, 1982). Let $\left( Y_{i},\delta_{i} \right)$ be the observed survival time and censoring indicator and let $X_{i}$ be the predictors for individual $i=1,...,n$. The predicted risk score, $\langle\psi,X_{i}\rangle$, is obtained by solving

$$\min_{\psi, \xi} \frac{1}{2}\left\| \psi\right\|^{2}+\gamma\sum_{\left( i,j \right) : Y_{i}<Y_{j}} v_{ij}\xi_{ij}$$

subject to $\left\langle\psi,X_{j} \right\rangle-\left\langle\psi,X_{i} \right\rangle\geq- \xi_{ij}$and $\xi_{ij}\geq0, i,j=1,...,n,$

where $\psi\in\mathbb{R}^{p}$, $\langle\cdot,\cdot\rangle$ denotes the inner product, $v_{ij}=\delta_{i}I\left( Y_{i}<Y_{j} \right)$ is a comparability indicator for individuals $i$ and $j, \xi_{ij}$ are pair-specific slack variables, and $\gamma>0$ is a regularization parameter.

## Random Survival Forests

Random survival forests aggregate predictions from individual trees generated over bootstrap resampled datasets trained on random subsets of the predictors (Ishwaran et al, 2011). First, we obtain ‘*B*’ bootstrap-resampled datasets with *n* observations (sampled with replacement) and randomly select *p*^′^ *< p* predictors for each tree to be trained on. Individual survival trees are then grown, and the *B* trees are combined by averaging over the survival predictions for each tree.

## Gradient Boosting

Gradient boosting creates an ensemble from a series of individual survival trees (Hothorn et al, 2006). At each step, $m=1,...,M$, the gradient boosting algorithm makes a new prediction based on the previous prediction, $F_{m}-1(\boldsymbol{X})$, and a new prediction from a single tree, $f_{m}(\boldsymbol{X})$, such that

$$F_{m}\left( \boldsymbol{X} \right)= F_{m}-1\left( \boldsymbol{X} \right)+ w_{m}f_{m}\left( \boldsymbol{X} \right),$$

where $0<w_{m}\leq1$ is the step size and the number of steps, $M$, can be tuned.

## Ensemble Averaging

Suppose there are $T$ base learners $\left\{ \phi_{1},\phi_{2},...,\phi_{T} \right\}$ (e.g., the aforementioned four prediction models) with corresponding predictions, *ϕ_t_*(*X*), based on predictors of *X*. Ensemble averaging combines these predictions by averaging their values, i.e.,

$$\frac{1}{T}\sum_{t=1}^{T} \phi_{t}\left( \boldsymbol{X} \right).$$

## Feature Importance

Feature importance measures the absolute decrease in C-index after we permute or “shuffle” the values of the concerned feature in the dataset, which effectively amounts to removal of the feature from the dataset while retaining the data structure (Breiman, 2001; Fisher et al, 2019). A feature is viewed as more important if the C-index decreases more after permutation, whereas it is considered unimportant if the C-index remains unchanged after permutation. An algorithm (Molnar, 2020) for computing the feature importance is given as follows.

**Algorithm 1** The permutation feature importance algorithm

**Input:** Based on an established model $f$ (e.g., a model constructed from the training data) and with features $\boldsymbol{X}\in\mathbb{R}^{n\times p}$ and outcome $y\in R^{n}$ from the testing data, compute C-index $c_{base}= c\left( y,f\left( \boldsymbol{X} \right) \right)$

**for** $j=1, 2, ...,p$ **do**

Permute values of feature $j$ only in the original feature matrix $\boldsymbol{X}$ to generate a permuted feature matrix ${\tilde{\boldsymbol{X}}}_{j}$, and calculate C-index $c_{j}=c\left( y,f\left( {\tilde{\boldsymbol{X}}}_{j} \right) \right)$

Calculate feature importance for feature $j$ as $FI_{j}=\max\left( 0,c_{base}-c_{j} \right)$

**end for**

# E. Additional Descriptive Results

**Table S3**. Descriptive statistics for candidate predictors, overall, and stratified by age and median number of comorbidity conditions.

| **Predictors** | **Overall,**  **N = 3,310^1^** | **Stratified by Age** |  | **Stratified by Comorbidities** |  |
| --- | --- | --- | --- | --- | --- |
|  |  | **≤ 65,**  **N = 1,976^1^** | **> 65,**  **N = 1,334^1^** | **≤ 7,**  **N = 1,679^1^** | **> 7,**  **N = 1,631^1^** |
| **Demographic Characteristics** |  |  |  |  |  |
| Age (years) | 61 (46, 73) | 50 (34, 58) | 75 (70, 82) | 55 (38, 68) | 66 (54, 76) |
| Sex |  |  |  |  |  |
| Female | 1,461 (44%) | 855 (43%) | 606 (45%) | 737 (44%) | 724 (44%) |
| Male | 1,849 (56%) | 1,121 (57%) | 728 (55%) | 942 (56%) | 907 (56%) |
| Race |  |  |  |  |  |
| African American | 692 (21%) | 475 (24%) | 217 (17%) | 327 (20%) | 365 (23%) |
| American Indian or Alaska Native | 20 (0.6%) | 11 (0.6%) | 9 (0.7%) | 7 (0.4%) | 13 (0.8%) |
| Asian | 88 (2.7%) | 51 (2.6%) | 37 (2.8%) | 55 (3.3%) | 33 (2.0%) |
| Caucasian | 2,230 (68%) | 1,269 (65%) | 961 (73%) | 1,140 (69%) | 1,090 (67%) |
| Native Hawaiian/Other Pacific Islander | 5 (0.2%) | 3 (0.2%) | 2 (0.2%) | 2 (0.1%) | 3 (0.2%) |
| Other | 179 (5.5%) | 111 (5.7%) | 68 (5.2%) | 92 (5.6%) | 87 (5.4%) |
| Patient Refused | 10 (0.3%) | 6 (0.3%) | 4 (0.3%) | 7 (0.4%) | 3 (0.2%) |
| Unknown | 49 (1.5%) | 32 (1.6%) | 17 (1.3%) | 25 (1.5%) | 24 (1.5%) |
| Ethnicity |  |  |  |  |  |
| Hispanic or Latino | 150 (4.6%) | 113 (5.8%) | 37 (2.8%) | 82 (5.0%) | 68 (4.2%) |
| Non-Hispanic or Latino | 3,045 (93%) | 1,803 (92%) | 1,242 (95%) | 1,527 (93%) | 1,518 (94%) |
| Patient Refused | 10 (0.3%) | 4 (0.2%) | 6 (0.5%) | 6 (0.4%) | 4 (0.2%) |
| Unknown | 55 (1.7%) | 31 (1.6%) | 24 (1.8%) | 30 (1.8%) | 25 (1.5%) |
| Body Mass Index (kg/m^2^) | 29 (25, 35) | 30 (25, 36) | 28 (25, 33) | 29 (24, 35) | 29 (25, 35) |
| **Comorbidity Conditions** |  |  |  |  |  |
| Alcohol Abuse | 382 (12%) | 264 (13%) | 118 (8.8%) | 80 (4.8%) | 302 (19%) |
| Blood Loss Anemia | 607 (18%) | 364 (18%) | 243 (18%) | 105 (6.3%) | 502 (31%) |
| Cardiac Arrhythmias | 2,305 (70%) | 1,292 (65%) | 1,013 (76%) | 835 (50%) | 1,470 (90%) |
| Chronic Pulmonary Disease | 1,491 (45%) | 859 (43%) | 632 (47%) | 473 (28%) | 1,018 (62%) |
| Coagulopathy | 1,137 (34%) | 676 (34%) | 461 (35%) | 280 (17%) | 857 (53%) |
| Congestive Heart Failure | 1,140 (34%) | 538 (27%) | 602 (45%) | 162 (9.6%) | 978 (60%) |
| Deficiency Anemia | 953 (29%) | 534 (27%) | 419 (31%) | 149 (8.9%) | 804 (49%) |
| Depression | 1,457 (44%) | 890 (45%) | 567 (43%) | 442 (26%) | 1,015 (62%) |
| Diabetes, Complicated | 1,201 (36%) | 594 (30%) | 607 (46%) | 248 (15%) | 953 (58%) |
| Diabetes, Uncomplicated | 1,336 (40%) | 673 (34%) | 663 (50%) | 332 (20%) | 1,004 (62%) |
| Drug Abuse | 553 (17%) | 405 (20%) | 148 (11%) | 134 (8.0%) | 419 (26%) |
| Fluid and Electrolyte Disorders | 2,312 (70%) | 1,317 (67%) | 995 (75%) | 829 (49%) | 1,483 (91%) |
| Hypertension, Complicated | 1,427 (43%) | 648 (33%) | 779 (58%) | 216 (13%) | 1,211 (74%) |
| Hypertension, Uncomplicated | 2,320 (70%) | 1,169 (59%) | 1,151 (86%) | 836 (50%) | 1,484 (91%) |
| Hypothyroidism | 771 (23%) | 378 (19%) | 393 (29%) | 206 (12%) | 565 (35%) |
| Liver Disease | 918 (28%) | 563 (28%) | 355 (27%) | 196 (12%) | 722 (44%) |
| Lymphoma | 299 (9.0%) | 171 (8.7%) | 128 (9.6%) | 55 (3.3%) | 244 (15%) |
| Metastatic Cancer | 749 (23%) | 337 (17%) | 412 (31%) | 173 (10%) | 576 (35%) |
| Obesity | 1,669 (50%) | 1,058 (54%) | 611 (46%) | 672 (40%) | 997 (61%) |
| Other Neurological Disorders | 997 (30%) | 529 (27%) | 468 (35%) | 255 (15%) | 742 (45%) |
| Paralysis | 393 (12%) | 260 (13%) | 133 (10.0%) | 72 (4.3%) | 321 (20%) |
| Peptic Ulcer Disease, Excluding Bleeding | 370 (11%) | 211 (11%) | 159 (12%) | 41 (2.4%) | 329 (20%) |
| Peripheral Vascular Disorders | 1,075 (32%) | 532 (27%) | 543 (41%) | 179 (11%) | 896 (55%) |
| Psychoses | 411 (12%) | 218 (11%) | 193 (14%) | 54 (3.2%) | 357 (22%) |
| Pulmonary Circulation Disorders | 886 (27%) | 514 (26%) | 372 (28%) | 164 (9.8%) | 722 (44%) |
| Renal Failure | 1,319 (40%) | 625 (32%) | 694 (52%) | 208 (12%) | 1,111 (68%) |
| Rheumatoid Arthritis, Collagen Vascular Diseases | 546 (16%) | 328 (17%) | 218 (16%) | 105 (6.3%) | 441 (27%) |
| Solid Tumor Without Metastasis | 749 (23%) | 344 (17%) | 405 (30%) | 192 (11%) | 557 (34%) |
| Valvular Disease | 758 (23%) | 379 (19%) | 379 (28%) | 118 (7.0%) | 640 (39%) |
| Weight Loss | 1,031 (31%) | 571 (29%) | 460 (34%) | 278 (17%) | 753 (46%) |
| **Physiologic Measurements** |  |  |  |  |  |
| Oxygen Saturation | 95.47 (93.97, 97.00) | 95.75 (94.07, 97.38) | 95.17 (93.70, 96.50) | 95.32 (93.76, 97.00) | 95.54 (94.11, 97.00) |
| Temperature (F) | 98.32 (98.00, 98.80) | 98.37 (98.05, 98.88) | 98.25 (97.94, 98.71) | 98.40 (98.07, 98.90) | 98.25 (97.97, 98.68) |
| Respiratory Rate | 18.8 (17.5, 21.5) | 19.0 (17.5, 22.0) | 18.6 (17.5, 21.0) | 19.0 (17.6, 22.0) | 18.5 (17.4, 21.1) |
| Diastolic Blood Pressure | 68 (62, 74) | 68 (62, 75) | 67 (62, 73) | 68 (63, 74) | 67 (62, 74) |
| Systolic Blood Pressure | 125 (113, 137) | 121 (110, 133) | 130 (118, 143) | 122 (112, 134) | 127 (115, 141) |
| Heart Rate | 82 (73, 93) | 85 (76, 96) | 78 (70, 88) | 83 (74, 93) | 82 (73, 92) |
| Smoking Status |  |  |  |  |  |
| Current | 203 (7.1%) | 156 (9.2%) | 47 (4.1%) | 110 (7.5%) | 93 (6.6%) |
| Former | 963 (34%) | 428 (25%) | 535 (46%) | 376 (26%) | 587 (42%) |
| Never | 1,473 (51%) | 973 (57%) | 500 (43%) | 822 (56%) | 651 (46%) |
| Unknown | 223 (7.8%) | 146 (8.6%) | 77 (6.6%) | 152 (10%) | 71 (5.1%) |
| Alcohol Use |  |  |  |  |  |
| Yes | 872 (30%) | 544 (32%) | 328 (28%) | 493 (34%) | 379 (27%) |
| No | 930 (32%) | 508 (30%) | 422 (36%) | 314 (22%) | 616 (44%) |
| Not Asked | 33 (1.2%) | 24 (1.4%) | 9 (0.8%) | 21 (1.4%) | 12 (0.9%) |
| Unknown | 1,027 (36%) | 627 (37%) | 400 (35%) | 632 (43%) | 395 (28%) |
| Drug Use |  |  |  |  |  |
| Yes | 178 (6.2%) | 149 (8.7%) | 29 (2.5%) | 91 (6.2%) | 87 (6.2%) |
| No | 1,422 (50%) | 757 (44%) | 665 (57%) | 537 (37%) | 885 (63%) |
| Not Asked | 47 (1.6%) | 37 (2.2%) | 10 (0.9%) | 29 (2.0%) | 18 (1.3%) |
| Unknown | 1,215 (42%) | 760 (45%) | 455 (39%) | 803 (55%) | 412 (29%) |
| Sexual Activity |  |  |  |  |  |
| Yes | 671 (23%) | 511 (30%) | 160 (14%) | 341 (23%) | 330 (24%) |
| Not Currently | 546 (19%) | 240 (14%) | 306 (26%) | 174 (12%) | 372 (27%) |
| Not Asked | 186 (6.5%) | 112 (6.6%) | 74 (6.4%) | 101 (6.9%) | 85 (6.1%) |
| Unknown | 1,459 (51%) | 840 (49%) | 619 (53%) | 844 (58%) | 615 (44%) |
| **Neighborhood Socioeconomic Status** |  |  |  |  |  |
| Affluence Quartile |  |  |  |  |  |
| 1 | 786 (25%) | 511 (27%) | 275 (22%) | 425 (24%) | 361 (26%) |
| 2 | 662 (21%) | 409 (22%) | 253 (20%) | 366 (21%) | 296 (22%) |
| 3 | 736 (24%) | 442 (24%) | 294 (23%) | 433 (25%) | 303 (22%) |
| 4 | 939 (30%) | 507 (27%) | 432 (34%) | 530 (30%) | 409 (30%) |
| Disadvantage Quartile |  |  |  |  |  |
| 1 | 948 (30%) | 534 (29%) | 414 (33%) | 559 (32%) | 389 (28%) |
| 2 | 742 (24%) | 416 (22%) | 326 (26%) | 432 (25%) | 310 (23%) |
| 3 | 652 (21%) | 417 (22%) | 235 (19%) | 341 (19%) | 311 (23%) |
| 4 | 781 (25%) | 502 (27%) | 279 (22%) | 422 (24%) | 359 (26%) |
| Ethnic Immigrant Quartile |  |  |  |  |  |
| 1 | 1,341 (43%) | 812 (43%) | 529 (42%) | 786 (45%) | 555 (41%) |
| 2 | 1,180 (38%) | 701 (38%) | 479 (38%) | 637 (36%) | 543 (40%) |
| 3 | 522 (17%) | 310 (17%) | 212 (17%) | 290 (17%) | 232 (17%) |
| 4 | 80 (2.6%) | 46 (2.5%) | 34 (2.7%) | 41 (2.3%) | 39 (2.8%) |
| Education Quartile |  |  |  |  |  |
| 1 | 1,100 (35%) | 625 (33%) | 475 (38%) | 632 (36%) | 468 (34%) |
| 2 | 972 (31%) | 578 (31%) | 394 (31%) | 540 (31%) | 432 (32%) |
| 3 | 766 (25%) | 482 (26%) | 284 (23%) | 426 (24%) | 340 (25%) |
| 4 | 285 (9.1%) | 184 (9.8%) | 101 (8.1%) | 156 (8.9%) | 129 (9.4%) |
| **Radiomic Features** |  |  |  |  |  |
| Dependence Non-Uniformity | -0.12 (-0.61, 0.49) | -0.17 (-0.66, 0.44) | -0.04 (-0.52, 0.55) | -0.17 (-0.66, 0.43) | -0.08 (-0.56, 0.58) |
| Large Area High Gray Level Emphasis | -0.36 (-0.56, -0.07) | -0.36 (-0.60, -0.08) | -0.34 (-0.50, -0.05) | -0.36 (-0.60, -0.10) | -0.36 (-0.52, -0.03) |
| Median | -0.61 (-0.61, 0.35) | -0.61 (-0.61, 0.20) | -0.61 (-0.61, 0.57) | -0.61 (-0.61, 0.09) | -0.58 (-0.61, 0.61) |
| Maximal Correlation Coefficient | 0.05 (-0.41, 0.59) | 0.05 (-0.44, 0.58) | 0.06 (-0.39, 0.60) | 0.02 (-0.45, 0.55) | 0.09 (-0.36, 0.63) |
| Robust Mean Absolute Deviation | -0.35 (-0.42, -0.07) | -0.34 (-0.42, -0.04) | -0.36 (-0.42, -0.11) | -0.31 (-0.41, -0.03) | -0.38 (-0.43, -0.11) |
| Zone Entropy | -0.34 (-0.62, 0.17) | -0.37 (-0.67, 0.11) | -0.29 (-0.56, 0.23) | -0.37 (-0.65, 0.06) | -0.30 (-0.60, 0.40) |
| Kurtosis | -0.47 (-0.58, 0.21) | -0.48 (-0.59, 0.24) | -0.45 (-0.58, 0.19) | -0.50 (-0.59, 0.10) | -0.43 (-0.58, 0.32) |

^1^Median (IQR); n (%)

# References

Abbas A, Abdelsamea MM, Gaber MM (2020) Classification of covid-19 in chest x-ray images using DeTraC deep convolutional neural network [published online ahead of print, 2020 sep 5]. Appl Intell (Dordr) pp 1–11

Akl EA, Blaˇzi´c I, Yaacoub S, et al (2021) Use of chest imaging in the diagnosis and management of covid-19: a who rapid advice guide. Radiology 298(2):E63–E69

Alqahtani JS, Oyelade T, Aldhahir AM, et al (2020) Prevalence, severity and mortality associated with copd and smoking in patients with covid-19: a rapid systematic review and meta-analysis. PloS One 15(5):e0233,147

Balbi M, Caroli A, Corsi A, et al (2021) Chest x-ray for predicting mortality and the need for ventilatory support in covid-19 patients presenting to the emergency department. Eur Radiol 31(4):1999–2012

Blain M, Kassin MT, Varble N, et al (2021) Determination of disease severity in covid-19 patients using deep learning in chest x-ray images. Diagn Interv Radiol 27(1):20–27

Borkowski AA, Viswanadhan NA, Thomas LB, et al (2020) Using artificial intelligence for covid-19 chest x-ray diagnosis. Fed Pract 37(9):398–404

Breiman L (2001) Random forests. Machine Learning 45(1):5–32

Castelli G, Semenzato U, Lococo S, et al (2022) Brief communication: Chest radiography score in young covid-19 patients: Does one size fit all? PloS one 17(2):e0264,172

Centers for Disease Control and Prevention (2022a) Cdc covid data tracker. https://covid.cdc.gov/covid-data-tracker/, accessed: 2022-06-13

Centers for Disease Control and Prevention (2022b) Scientific evidence for conditions associated with higher risk for severe covid-19. https://www.cdc.gov/coronavirus/2019-ncov/science/sciencebriefs/underlying-evidence-table.html, accessed: 2022-06-13

Chandra TB, Verma K, Singh BK, et al (2021) Coronavirus disease (covid19) detection in chest x-ray images using majority voting based classifier ensemble. Expert Syst Appl 165:113,909

Chu A, Sehgal CM, Greenleaf JF (1990) Use of gray value distribution of run lengths for texture analysis. Pattern Recognition Letters 11(6):415–419

Clarke P, Melendez R (Ann Arbor, MI) National neighborhood data archive (nanda): Neighborhood socioeconomic and demographic characteristics of census tracts, united states, 2000-2010. Inter-university Consortium for Political and Social Research

Cox DR (1972) Regression models and life tables (with discussion). Journal of the Royal Statistical Society, Series B 34(2):187–220

Crabb BT, Lyons A, Bale M, et al (2020) Comparison of international classification of diseases and related health problems, tenth revision codes with electronic medical records among patients with symptoms of coronavirus disease 2019. JAMA Netw Open 3(8):e2017,703

Ebinger JE, Achamallah N, Ji H, et al (2020) Pre-existing traits associated with covid-19 illness severity. PloS One 15(7):e0236,240

Elixhauser A, Steiner C, Harris DR, et al (1998) Comorbidity measures for use with administrative data. Med Care 36(1):8–27

Fisher A, Rudin C, Dominici F (2019) All models are wrong, but many are useful: Learning a variable’s importance by studying an entire class of prediction models simultaneously. J Mach Learn Res 20(177):1–81

Galloway MM (1975) Texture analysis using gray level run lengths. Computer Graphics and Image Processing 4(2):172–179

Garrafa E, Vezzoli M, Ravanelli M, et al (2021) Early prediction of in-hospital death of covid-19 patients: a machine-learning model based on age, blood analyses, and chest x-ray score. Elife 10:e70,640

Gu T, Mack JA, Salvatore M, et al (2020) Characteristics associated with racial/ethnic disparities in covid-19 outcomes in an academic health care system. JAMA Netw Open 3(10):e2025,197

Gupta A, Madhavan MV, Poterucha TJ, et al (2021) Association between antecedent statin use and decreased mortality in hospitalized patients with COVID-19. Nat Commun 12(1):1325

Haralick RM, Shanmugam K, Dinstein I (1973) Textural features for image classification. IEEE Transactions on Systems, Man, and Cybernetics SMC3(6):610–621

Harrell FE, Califf RM, Pryor DB, et al (1982) Evaluating the yield of medical tests. JAMA 247(18):2543–2546

Harrell Jr FE, Lee KL, Mark DB (1996) Multivariable prognostic models: issues in developing models, evaluating assumptions and adequacy, and measuring and reducing errors. Statistics in medicine 15(4):361–387

He K, Zhang X, Ren S, et al (2016) Deep residual learning for image recognition. In: Proceedings of the IEEE Conference on Computer Vision and Pattern Recognition, pp 770–778

Hothorn T, Bühlmann P, Dudoit S, et al (2006) Survival ensembles. Biostatistics 7(3):355–373

Hussain L, Nguyen T, Li H, et al (2020) Machine-learning classification of texture features of portable chest x-ray accurately classifies covid-19 lung infection. Biomed Eng Online 19(1):88

Ishwaran H, Kogalur UB, Blackstone EH, et al (2008) Random survival forests. The Annals of Applied Statistics 2(3):841–860

Ishwaran H, Kogalur UB, Chen X, et al (2011) Random survival forests for high-dimensional data. Statistical Analysis and Data Mining: The ASA Data Science Journal 4(1):115–132

Ismael AM, Şengür A (2021) Deep learning approaches for covid-19 detection based on chest x-ray images. Expert Syst Appl 164:114,054

Jacobi A, Chung M, Bernheim A, et al (2020) Portable chest x-ray in coronavirus disease-19 (covid-19): A pictorial review. Clin Imaging 64:35–42

Jain AK (1989) Fundamentals of Digital Image Processing. Prentice-Hall, Inc

Jiao Z, Choi JW, Halsey K, et al (2021) Prognostication of patients with covid-19 using artificial intelligence based on chest x-rays and clinical data: a retrospective study. The Lancet Digital Health 3(5):e286–e294

Johri S, Goyal M, Jain S, et al (2021) A novel machine learning-based analytical framework for automatic detection of covid-19 using chest x-ray images. International Journal of Imaging Systems and Technology 31(3):1105–1119

Jordan RE, Adab P, Cheng KK (2020) Covid-19: risk factors for severe disease and death. BMJ 368:m1198

Karim SSA, Karim QA (2021) Omicron sars-cov-2 variant: a new chapter in the covid-19 pandemic. The Lancet 398(10317):2126–2128

Khan MMA, Khan MN, Mustagir MG, et al (2020) Effects of underlying morbidities on the occurrence of deaths in covid-19 patients: A systematic review and meta-analysis. J Glob Health 10(2):020,503

Kim HW, Capaccione KM, Li G, et al (2020) The role of initial chest x-ray in triaging patients with suspected covid-19 during the pandemic. Emerg Radiol 27(6):617–621

Kim L, Garg S, O’Halloran A, et al (2021) Risk factors for intensive care unit admission and in-hospital mortality among hospitalized adults identified through the us coronavirus disease 2019 (covid-19)-associated hospitalization surveillance network (covid-net). Clin Infect Dis 72(9):e206–e214

Krizhevsky A, Sutskever I, Hinton GE (2012) Imagenet classification with deep convolutional neural networks. Advances in Neural Information Processing Systems 25:1–9

Kupferschmidt K, Wadman M (2021) Delta variant triggers new phase in the pandemic. Science 372(6549):1375–1376

Liu X, Zhou H, Zhou Y, et al (2020) Temporal radiographic changes in covid-19 patients: relationship to disease severity and viral clearance. Sci Rep 10(1):10,263

Maroldi R, Rondi P, Agazzi GM, et al (2021) Which role for chest x-ray score in predicting the outcome in covid-19 pneumonia? Eur Radiol 31(6):4016–4022

McCue C, Cowan R, Quasim T, et al (2021) Long term outcomes of critically ill covid-19 pneumonia patients: early learning. Intensive Care Med 47(2):240– 241

Michelen M, Cheng V, Manoharan L, et al (2021) Characterising long term covid-19: a living systematic review. BMJ Glob Health 6(9):e005,427

Mikami T, Miyashita H, Yamada T, et al (2021) Risk factors for mortality in patients with covid-19 in new york city. J Gen Intern Med 36(1):17–26

Molnar C (2020) Interpretable machine learning. Lulu. com

Monaco CG, Zaottini F, Schiaffino S, et al (2020) Chest x-ray severity score in covid-19 patients on emergency department admission: a two-centre study. Eur Radiol Exp 4(1):68

Nypaver M, Macy M, Pribble J, et al (2018) The michigan emergency department improvement collaborative: A novel model for implementing large scale practice change in pediatric emergency care. Pediatrics 142(1 MeetingAbstract):105–105

Pölsterl S, Navab N, Katouzian A (Preprint posted online November 21, 2016) An efficient training algorithm for kernel survival support vector machines. arXiv ArXiv:1611.07054 [cs.LG]. doi: 10.48550/arXiv.1611.07054

Quan H, Sundararajan V, Halfon P, et al (2005) Coding algorithms for defining comorbidities in icd-9-cm and icd-10 administrative data. Med Care 43(11):1130–1139

Rod JE, Oviedo-Trespalacios O, Cortes-Ramirez J (2020) A brief-review of the risk factors for COVID-19 severity. Rev Saude Publica 54:60

Rosenthal N, Cao Z, Gundrum J, et al (2020) Risk factors associated with inhospital mortality in a us national sample of patients with covid-19. JAMA Netw Open 3(12):e2029,058

Salerno S, Li Y (Preprint posted online May 5, 2022) High-dimensional survival analysis: Methods and applications. arXiv ArXiv:2205.02948 [stat.ME]. doi: 10.48550/arXiv.2205.02948

Salerno S, Sun Y, Morris EL, et al (2021a) Comprehensive evaluation of covid-19 patient short-and long-term outcomes: Disparities in healthcare utilization and post-hospitalization outcomes. Plos one 16(10):e0258,278

Salerno S, Zhao Z, Prabhu Sankar S, et al (2021b) Patterns of repeated diagnostic testing for covid-19 in relation to patient characteristics and outcomes. J Intern Med 289(5):726–737

Salvatore M, Gu T, Mack JA, et al (2021) A phenome-wide association study (phewas) of covid-19 outcomes by race using the electronic health records data in michigan medicine. J Clin Med 10(7):1351

Schalekamp S, Huisman M, van Dijk RA, et al (2021) Model-based prediction of critical illness in hospitalized patients with covid-19. Radiology 298(1):E46–E54

Selvi JT, Subhashini K, Methini M (2021) Investigation of covid-19 chest x-ray images using texture features–a comprehensive approach. In: Computational

Modelling and Imaging for SARS-CoV-2 and COVID-19. CRC Press, p 45– 58

Shen B, Hoshmand-Kochi M, Abbasi A, et al (2021) Initial chest radiograph scores inform covid-19 status, intensive care unit admission and need for mechanical ventilation. Clin Radiol 76(6):473.e1–473.e7

Soda P, D’Amico NC, Tessadori J, et al (2021) Aiforcovid: predicting the clinical outcomes in patients with covid-19 applying ai to chest-x-rays. an italian multicentre study. Med Image Anal 74:102,216

Spector-Bagdady K, Higgins PD, Aaronson KD, et al (2020) Coronavirus disease 2019 (covid-19) clinical trial oversight at a major academic medical center: Approach of michigan medicine. Clin Infect Dis 71(16):2187–2190

Ssentongo P, Ssentongo AE, Heilbrunn ES, et al (2020) Association of cardiovascular disease and 10 other pre-existing comorbidities with covid-19 mortality: A systematic review and meta-analysis. PloS One 15(8):e0238,215

Therneau TM, Grambsch PM (2000) Modeling Survival Data: Extending the Cox Model , Springer, chap The Cox model, pp 39–77

Thibault G, Fertil B, Navarro C, et al (2013) Shape and texture indexes application to cell nuclei classification. International Journal of Pattern Recognition and Artificial Intelligence 27(01):1357,002

Uno H, Cai T, Tian L, et al (2007) Evaluating prediction rules for t-year survivors with censored regression models. Journal of the American Statistical Association 102(478):527–537

Uno H, Cai T, Pencina MJ, et al (2011) On the c-statistics for evaluating overall adequacy of risk prediction procedures with censored survival data. Stat Med 30(10):1105–1117

Van Belle V, Pelckmans K, Suykens J, et al (2007) Support vector machines for survival analysis. In: Proceedings of the Third International Conference on Computational Intelligence in Medicine and Healthcare (CIMED2007), pp 1–8

van Griethuysen JJM, Fedorov A, Parmar C, et al (2017) Computational radiomics system to decode the radiographic phenotype. Cancer Res 77(21):e104–e107

van Walraven C, Austin PC, Jennings A, et al (2009) A modification of the elixhauser comorbidity measures into a point system for hospital death using administrative data. Med Care 47(6):626–633

Varghese BA, Shin H, Desai B, et al (2021) Predicting clinical outcomes in covid-19 using radiomics on chest radiographs. Br J Radiol 94(1126):20210,221

Wang B, Li R, Lu Z, et al (2020) Does comorbidity increase the risk of patients with covid-19: evidence from meta-analysis. Aging (Albany NY) 12(7):6049– 6057

Williamson EJ, Walker AJ, Bhaskaran K, et al (2020) Factors associated with covid-19-related death using opensafely. Nature 584(7821):430–436

Wu Z, McGoogan JM (2020) Characteristics of and important lessons from the coronavirus disease 2019 (covid-19) outbreak in china: summary of a report of 72314 cases from the chinese center for disease control and prevention. JAMA 323(13):1239–1242

Yang J, Zheng Y, Gou X, et al (2020) Prevalence of comorbidities and its effects in patients infected with sars-cov-2: a systematic review and meta-analysis. Int J Infect Dis 94:91–95

Yasin R, Gouda W (2020) Chest x-ray findings monitoring covid-19 disease course and severity. The Egyptian Journal of Radiology and Nuclear Medicine 51(1):193

Yip SS, Aerts HJ (2016) Applications and limitations of radiomics. Physics in Medicine & Biology 61(13):R150

Zhang Q, Wu YN, Zhu SC (2018) Interpretable convolutional neural networks. In: Proceedings of the IEEE Conference on Computer Vision and Pattern Recognition, pp 8827–8836

Zhou ZH (2012) Ensemble methods: foundations and algorithms. CRC press

Zwanenburg A, Leger S, Valli`eres M, et al (Preprint posted online December 21, 2016) Image biomarker standardisation initiative. arXiv ArXiv:1612.07003 [cs.CV]. doi: 10.48550/arXiv.1612.07003
